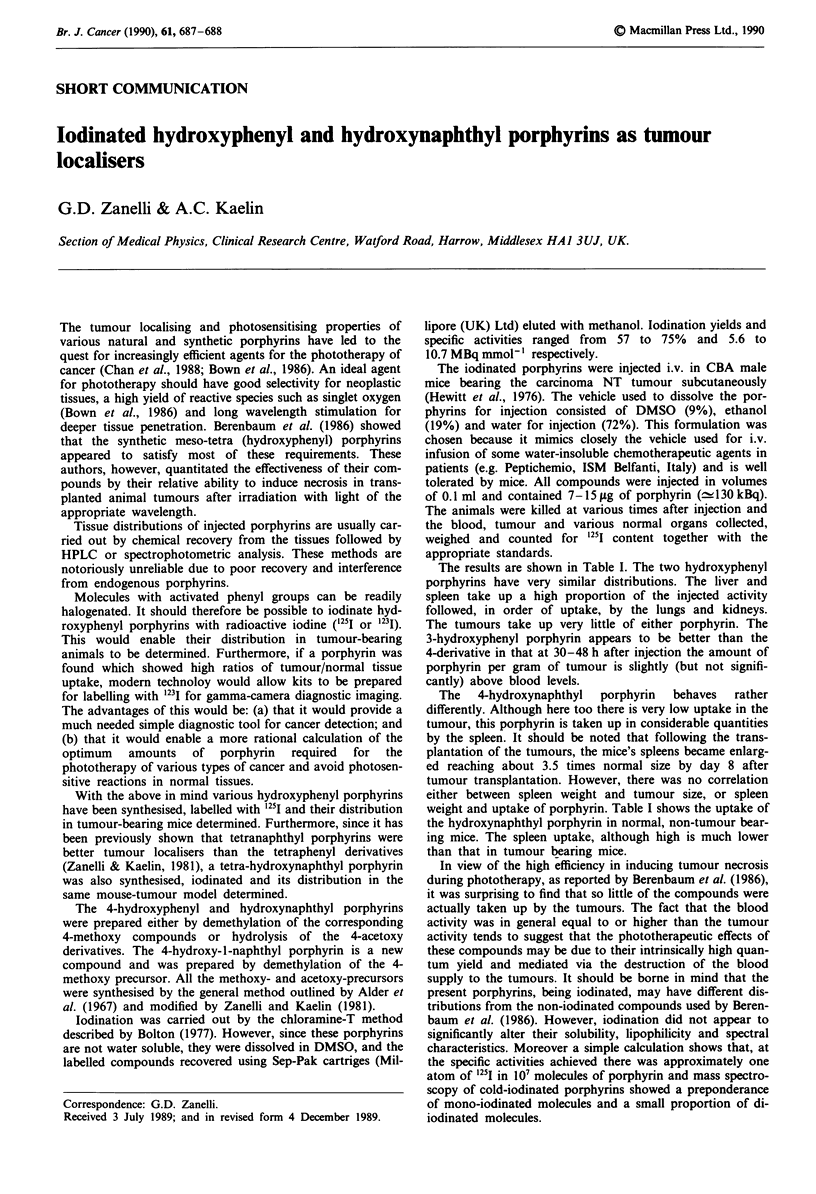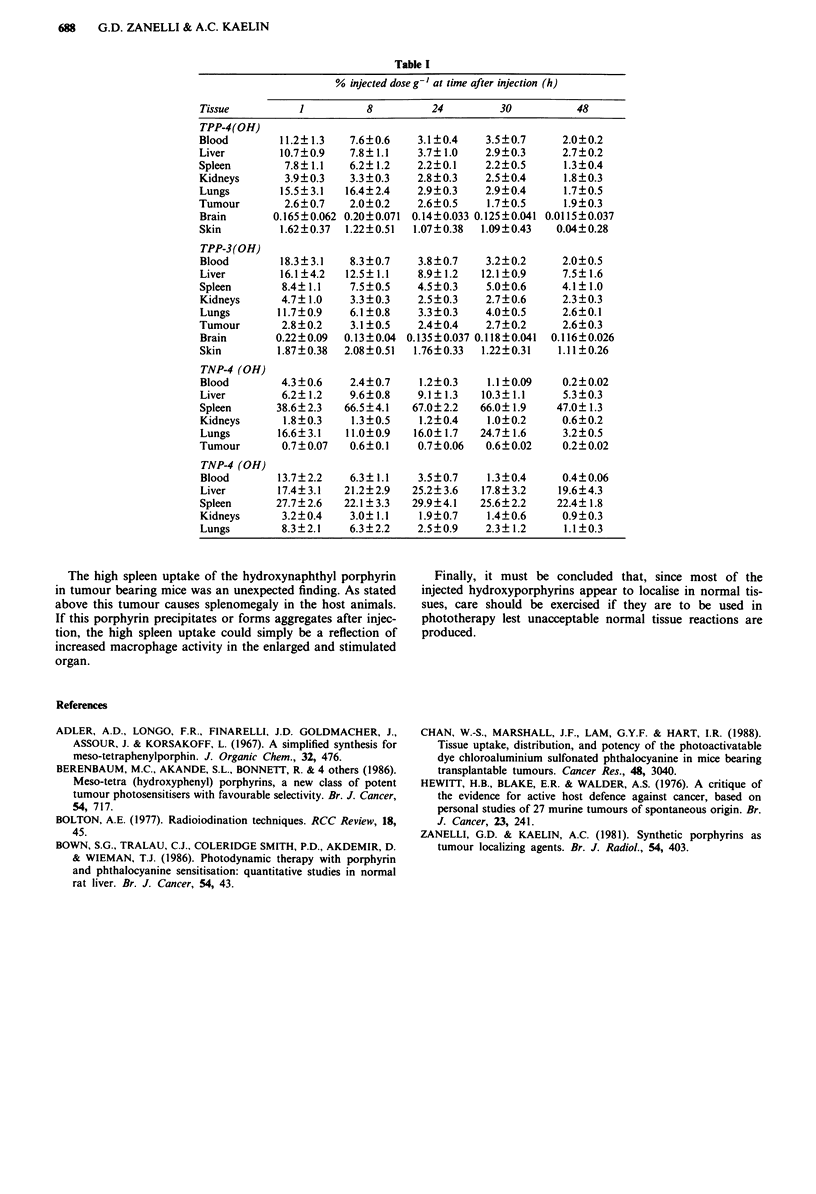# Iodinated hydroxyphenyl and hydroxynaphthyl porphyrins as tumour localisers.

**DOI:** 10.1038/bjc.1990.155

**Published:** 1990-05

**Authors:** G. D. Zanelli, A. C. Kaelin

**Affiliations:** Section of Medical Physics, Clinical Research Centre, Harrow, Middlesex, UK.


 © Macmillan Press Ltd., 1990

SHORT COMMUNICATION

### Iodinated hydroxyphenyl and hydroxynaphthyl porphyrins as tumour localisers

G.D. Zanelli & A.C. Kaelin

*Section of Medical Physics, Clinical Research Centre, Watford Road, Harrow, Middlesex HA1 3UJ, UK.*

The tumour localising and photosensitising properties of various natural and synthetic porphyrins have led to the quest for increasingly efficient agents for the phototherapy of cancer (Chan *et al.*, 1988; Bown *et al.*, 1986). An ideal agent for phototherapy should have good selectivity for neoplastic tissues, a high yield of reactive species such as singlet oxygen (Bown *et al.*, 1986) and long wavelength stimulation for deeper tissue penetration. Berenbaum *et al.* (1986) showed that the synthetic meso-tetra (hydroxyphenyl) porphyrins appeared to satisfy most of these requirements. These authors, however, quantitated the effectiveness of their compounds by their relative ability to induce necrosis in transplanted animal tumours after irradiation with light of the appropriate wavelength.

Tissue distributions of injected porphyrins are usually carried out by chemical recovery from the tissues followed by HPLC or spectrophotometric analysis. These methods are notoriously unreliable due to poor recovery and interference from endogenous porphyrins.

Molecules with activated phenyl groups can be readily halogenated. It should therefore be possible to iodinate hydroxyphenyl porphyrins with radioactive iodine ($^{125}I$ or $^{123}I$). This would enable their distribution in tumour-bearing animals to be determined. Furthermore, if a porphyrin was found which showed high ratios of tumour/normal tissue uptake, modern technoloy would allow kits to be prepared for labelling with $^{123}I$ for gamma-camera diagnostic imaging. The advantages of this would be: (a) that it would provide a much needed simple diagnostic tool for cancer detection; and (b) that it would enable a more rational calculation of the optimum amounts of porphyrin required for the phototherapy of various types of cancer and avoid photosensitive reactions in normal tissues.

With the above in mind various hydroxyphenyl porphyrins have been synthesised, labelled with $^{125}I$ and their distribution in tumour-bearing mice determined. Furthermore, since it has been previously shown that tetranaphthyl porphyrins were better tumour localisers than the tetraphenyl derivatives (Zanelli & Kaelin, 1981), a tetra-hydroxynaphthyl porphyrin was also synthesised, iodinated and its distribution in the same mouse-tumour model determined.

The 4-hydroxyphenyl and hydroxynaphthyl porphyrins were prepared either by demethylation of the corresponding 4-methoxy compounds or hydrolysis of the 4-acetoxy derivatives. The 4-hydroxy-1-naphthyl porphyrin is a new compound and was prepared by demethylation of the 4-methoxy precursor. All the methoxy- and acetoxy-precursors were synthesised by the general method outlined by Alder *et al.* (1967) and modified by Zanelli and Kaelin (1981).

Iodination was carried out by the chloramine-T method described by Bolton (1977). However, since these porphyrins are not water soluble, they were dissolved in DMSO, and the labelled compounds recovered using Sep-Pak cartriges (Millipore (UK) Ltd) eluted with methanol. Iodination yields and specific activities ranged from 57 to 75% and 5.6 to 10.7 MBq $mmol^{-1}$ respectively.

The iodinated porphyrins were injected i.v. in CBA male mice bearing the carcinoma NT tumour subcutaneously (Hewitt *et al.*, 1976). The vehicle used to dissolve the porphyrins for injection consisted of DMSO (9%), ethanol (19%) and water for injection (72%). This formulation was chosen because it mimics closely the vehicle used for i.v. infusion of some water-insoluble chemotherapeutic agents in patients (e.g. Peptichemio, ISM Belfanti, Italy) and is well tolerated by mice. All compounds were injected in volumes of 0.1 ml and contained 7–15 $\mu g$ of porphyrin ($\simeq$130 kBq). The animals were killed at various times after injection and the blood, tumour and various normal organs collected, weighed and counted for $^{125}I$ content together with the appropriate standards.

The results are shown in Table I. The two hydroxyphenyl porphyrins have very similar distributions. The liver and spleen take up a high proportion of the injected activity followed, in order of uptake, by the lungs and kidneys. The tumours take up very little of either porphyrin. The 3-hydroxyphenyl porphyrin appears to be better than the 4-derivative in that at 30–48 h after injection the amount of porphyrin per gram of tumour is slightly (but not significantly) above blood levels.

The 4-hydroxynaphthyl porphyrin behaves rather differently. Although here too there is very low uptake in the tumour, this porphyrin is taken up in considerable quantities by the spleen. It should be noted that following the transplantation of the tumours, the mice's spleens became enlarged reaching about 3.5 times normal size by day 8 after tumour transplantation. However, there was no correlation either between spleen weight and tumour size, or spleen weight and uptake of porphyrin. Table I shows the uptake of the hydroxynaphthyl porphyrin in normal, non-tumour bearing mice. The spleen uptake, although high is much lower than that in tumour bearing mice.

In view of the high efficiency in inducing tumour necrosis during phototherapy, as reported by Berenbaum *et al.* (1986), it was surprising to find that so little of the compounds were actually taken up by the tumours. The fact that the blood activity was in general equal to or higher than the tumour activity tends to suggest that the phototherapeutic effects of these compounds may be due to their intrinsically high quantum yield and mediated via the destruction of the blood supply to the tumours. It should be borne in mind that the present porphyrins, being iodinated, may have different distributions from the non-iodinated compounds used by Berenbaum *et al.* (1986). However, iodination did not appear to significantly alter their solubility, lipophilicity and spectral characteristics. Moreover a simple calculation shows that, at the specific activities achieved there was approximately one atom of $^{125}I$ in $10^7$ molecules of porphyrin and mass spectroscopy of cold-iodinated porphyrins showed a preponderance of mono-iodinated molecules and a small proportion of di-iodinated molecules.

Correspondence: G.D. Zanelli.
Received 3 July 1989; and in revised form 4 December 1989.

**Table I**

| Tissue | % injected dose $g^{-1}$ at time after injection (h) 1 | 8 | 24 | 30 | 48 |
|---|---|---|---|---|---|
| *TPP-4(OH)* | | | | | |
| Blood | 11.2±1.3 | 7.6±0.6 | 3.1±0.4 | 3.5±0.7 | 2.0±0.2 |
| Liver | 10.7±0.9 | 7.8±1.1 | 3.7±1.0 | 2.9±0.3 | 2.7±0.2 |
| Spleen | 7.8±1.1 | 6.2±1.2 | 2.2±0.1 | 2.2±0.5 | 1.3±0.4 |
| Kidneys | 3.9±0.3 | 3.3±0.3 | 2.8±0.3 | 2.5±0.4 | 1.8±0.3 |
| Lungs | 15.5±3.1 | 16.4±2.4 | 2.9±0.3 | 2.9±0.4 | 1.7±0.5 |
| Tumour | 2.6±0.7 | 2.0±0.2 | 2.6±0.5 | 1.7±0.5 | 1.9±0.3 |
| Brain | 0.165±0.062 | 0.20±0.071 | 0.14±0.033 | 0.125±0.041 | 0.0115±0.037 |
| Skin | 1.62±0.37 | 1.22±0.51 | 1.07±0.38 | 1.09±0.43 | 0.04±0.28 |
| *TPP-3(OH)* | | | | | |
| Blood | 18.3±3.1 | 8.3±0.7 | 3.8±0.7 | 3.2±0.2 | 2.0±0.5 |
| Liver | 16.1±4.2 | 12.5±1.1 | 8.9±1.2 | 12.1±0.9 | 7.5±1.6 |
| Spleen | 8.4±1.1 | 7.5±0.5 | 4.5±0.3 | 5.0±0.6 | 4.1±1.0 |
| Kidneys | 4.7±1.0 | 3.3±0.3 | 2.5±0.3 | 2.7±0.6 | 2.3±0.3 |
| Lungs | 11.7±0.9 | 6.1±0.8 | 3.3±0.3 | 4.0±0.5 | 2.6±0.1 |
| Tumour | 2.8±0.2 | 3.1±0.5 | 2.4±0.4 | 2.7±0.2 | 2.6±0.3 |
| Brain | 0.22±0.09 | 0.13±0.04 | 0.135±0.037 | 0.118±0.041 | 0.116±0.026 |
| Skin | 1.87±0.38 | 2.08±0.51 | 1.76±0.33 | 1.22±0.31 | 1.11±0.26 |
| *TNP-4 (OH)* | | | | | |
| Blood | 4.3±0.6 | 2.4±0.7 | 1.2±0.3 | 1.1±0.09 | 0.2±0.02 |
| Liver | 6.2±1.2 | 9.6±0.8 | 9.1±1.3 | 10.3±1.1 | 5.3±0.3 |
| Spleen | 38.6±2.3 | 66.5±4.1 | 67.0±2.2 | 66.0±1.9 | 47.0±1.3 |
| Kidneys | 1.8±0.3 | 1.3±0.5 | 1.2±0.4 | 1.0±0.2 | 0.6±0.2 |
| Lungs | 16.6±3.1 | 11.0±0.9 | 16.0±1.7 | 24.7±1.6 | 3.2±0.5 |
| Tumour | 0.7±0.07 | 0.6±0.1 | 0.7±0.06 | 0.6±0.02 | 0.2±0.02 |
| *TNP-4 (OH)* | | | | | |
| Blood | 13.7±2.2 | 6.3±1.1 | 3.5±0.7 | 1.3±0.4 | 0.4±0.06 |
| Liver | 17.4±3.1 | 21.2±2.9 | 25.2±3.6 | 17.8±3.2 | 19.6±4.3 |
| Spleen | 27.7±2.6 | 22.1±3.3 | 29.9±4.1 | 25.6±2.2 | 22.4±1.8 |
| Kidneys | 3.2±0.4 | 3.0±1.1 | 1.9±0.7 | 1.4±0.6 | 0.9±0.3 |
| Lungs | 8.3±2.1 | 6.3±2.2 | 2.5±0.9 | 2.3±1.2 | 1.1±0.3 |

The high spleen uptake of the hydroxynaphthyl porphyrin in tumour bearing mice was an unexpected finding. As stated above this tumour causes splenomegaly in the host animals. If this porphyrin precipitates or forms aggregates after injection, the high spleen uptake could simply be a reflection of increased macrophage activity in the enlarged and stimulated organ.

Finally, it must be concluded that, since most of the injected hydroxyporphyrins appear to localise in normal tissues, care should be exercised if they are to be used in phototherapy lest unacceptable normal tissue reactions are produced.

### References

ADLER, A.D., LONGO, F.R., FINARELLI, J.D. GOLDMACHER, J., ASSOUR, J. & KORSAKOFF, L. (1967). A simplified synthesis for meso-tetraphenylporphin. *J. Organic Chem.*, **32**, 476.

BERENBAUM, M.C., AKANDE, S.L., BONNETT, R. & 4 others (1986). Meso-tetra (hydroxyphenyl) porphyrins, a new class of potent tumour photosensitisers with favourable selectivity. *Br. J. Cancer*, **54**, 717.

BOLTON, A.E. (1977). Radioiodination techniques. *RCC Review*, **18**, 45.

BOWN, S.G., TRALAU, C.J., COLERIDGE SMITH, P.D., AKDEMIR, D. & WIEMAN, T.J. (1986). Photodynamic therapy with porphyrin and phthalocyanine sensitisation: quantitative studies in normal rat liver. *Br. J. Cancer*, **54**, 43.

CHAN, W.-S., MARSHALL, J.F., LAM, G.Y.F. & HART, I.R. (1988). Tissue uptake, distribution, and potency of the photoactivatable dye chloroaluminium sulfonated phthalocyanine in mice bearing transplantable tumours. *Cancer Res.*, **48**, 3040.

HEWITT, H.B., BLAKE, E.R. & WALDER, A.S. (1976). A critique of the evidence for active host defence against cancer, based on personal studies of 27 murine tumours of spontaneous origin. *Br. J. Cancer*, **23**, 241.

ZANELLI, G.D. & KAELIN, A.C. (1981). Synthetic porphyrins as tumour localizing agents. *Br. J. Radiol.*, **54**, 403.